# Community‐level socioeconomic distress is associated with nutritional status in adults with sickle cell anemia

**DOI:** 10.1002/jha2.661

**Published:** 2023-02-17

**Authors:** Syeda Akila Ally, Jin Han, Ryan Sun, Robert E. Molokie, Victor R. Gordeuk, James P. Lash, Santosh L. Saraf

**Affiliations:** ^1^ Division of Hematology and Oncology Department of Medicine University of Illinois at Chicago Chicago Illinois USA; ^2^ Department of Pharmacy Practice College of Pharmacy University of Illinois at Chicago Chicago Illinois USA; ^3^ Department of Medicine Jesse Brown VA Medical Center Chicago Illinois USA; ^4^ Division of Nephrology Department of Medicine University of Illinois at Chicago Chicago Illinois USA

**Keywords:** distressed community index, nutrition, sickle cell anemia, socioeconomic

## Abstract

Sickle cell anemia (SCA) negatively impacts the ability to achieve educational and occupational goals increasing vulnerability to socioeconomic challenges. In a cross‐sectional analysis of 332 SCA adults, we investigated whether the distressed community index (DCI) was associated with SCA‐related complications and nutritional status. More patients with higher DCI had Medicaid insurance. A higher DCI was independently associated with tobacco use and lower body mass index, serum albumin, and vitamin D 25‐OH levels after adjusting for insurance status but was not associated with SCA‐related complications. Future studies investigating access to healthy foods may help improve health equity in patients with SCA.

## 1 INTRODUCTION

Sickle cell anemia (SCA) is among the most common inherited red blood cell disorders affecting approximately one in 500 African Americans and 25 million people worldwide [1]. Hallmark features of SCA include vaso‐occlusion and intravascular hemolysis, which lead to a myriad of acute and chronic complications, including vaso‐occlusive pain episodes (VOE), acute chest syndrome, and stroke. These complications negatively impact the ability of people with SCA to achieve educational and occupational goals [2, 3]. In addition, African Americans are disproportionately affected by socioeconomic inequity making people with SCA a particularly vulnerable population to social determinants of health.

The Distressed Communities Index (DCI) is a tool that was developed to compare the economic well‐being of US communities by zip code. The components of the DCI score include measures of educational level, housing vacancy, unemployment, poverty rate, and median income. A study which assessed the impact of community‐level socioeconomic well‐being using the DCI found that Medicare beneficiaries living in distressed communities were at greater risk for lower health care quality, including annual wellness checks and receiving recommended annual tests, and experienced a higher rate of potentially avoidable hospital admissions, 30‐day hospital readmissions, and higher overall mortality [4]. Community‐level socioeconomic distress can also reduce access to healthy foods and increase metabolic risk factors for morbidity and early mortality [5].

The impact of community level socioeconomic distress in patients with SCA is less clear. In one cohort from the United Kingdom, people with SCA living in disadvantaged neighborhoods had higher rates of emergency room readmissions and inpatient mortality compared to those living in less disadvantaged neighborhoods [6]. In another study, children with SCA living in socioeconomically deprived neighborhoods had less episodes of recurrent acute chest syndrome compared to those living in more affluent neighborhoods [7].

The purpose of this cross‐sectional study was to determine (1) the prevalence of community level socioeconomic distress, as determined by the DCI, and (2) its association with SCA‐related complications and markers of nutritional status in adults with SCA.

## 2 METHODS

The study was approved by the University of Illinois Chicago Institution Review Board and subjects provided written informed consent in accordance with the Declaration of Helsinki. Between August 2010 and June 2018, 332 adults with SCA (genotype hemoglobin SS or Sβ^0^) were recruited into a registry during a routine clinic visit. Laboratory and clinical parameters, including insurance status, were collected either during that outpatient clinic visit or from an outpatient clinic visit closest to the time of recruitment. VOE frequency was determined by the number of VOE requiring medical attention in the emergency room, acute care center, or hospitalization in the 12 months preceding enrolment.

The DCI is a composite of seven socioeconomic indicators (no high school diploma, housing vacancy rate, adults not working, poverty rate, median income ratio, and changes in employment and establishments) that estimate the economic well‐being of a community [4, 8]. The DCI is maintained by Economic Innovation Group (website eig.org/dci) and is built from the US Census Bureau's American Community Survey and the Census Bureau's Business Patterns datasets from 2016–2020. DCI score ranges from 0 to 100 with a higher score indicating a more distressed community. The DCI score was determined for each subject using their home address zip code listed in the electronic medical record system.

We compared SCA‐related complications by insurance status using the test for linear trend and Cochran's test for linear trend. Comparisons of linear and categorical variables by DCI tertile (low: DCI < 73; middle: DCI 73 – 95; high: DCI > 95) were conducted using the test for linear trend and Cochran's test for linear trend, respectively. The associations of DCI with SCA‐related complications, tobacco use, and markers of nutritional status (body mass index, serum albumin, vitamin D 25‐OH) were assessed by linear or logistic regression analysis, adjusting for age, sex, hydroxyurea use, and insurance type. The beta coefficients and odds ratios (OR) and 95% confidence intervals (CIs) that are provided are based on increments of 10 for DCI. Median and interquartile ranges (IQR) are provided.

## 3 RESULTS

**FIGURE 1:**
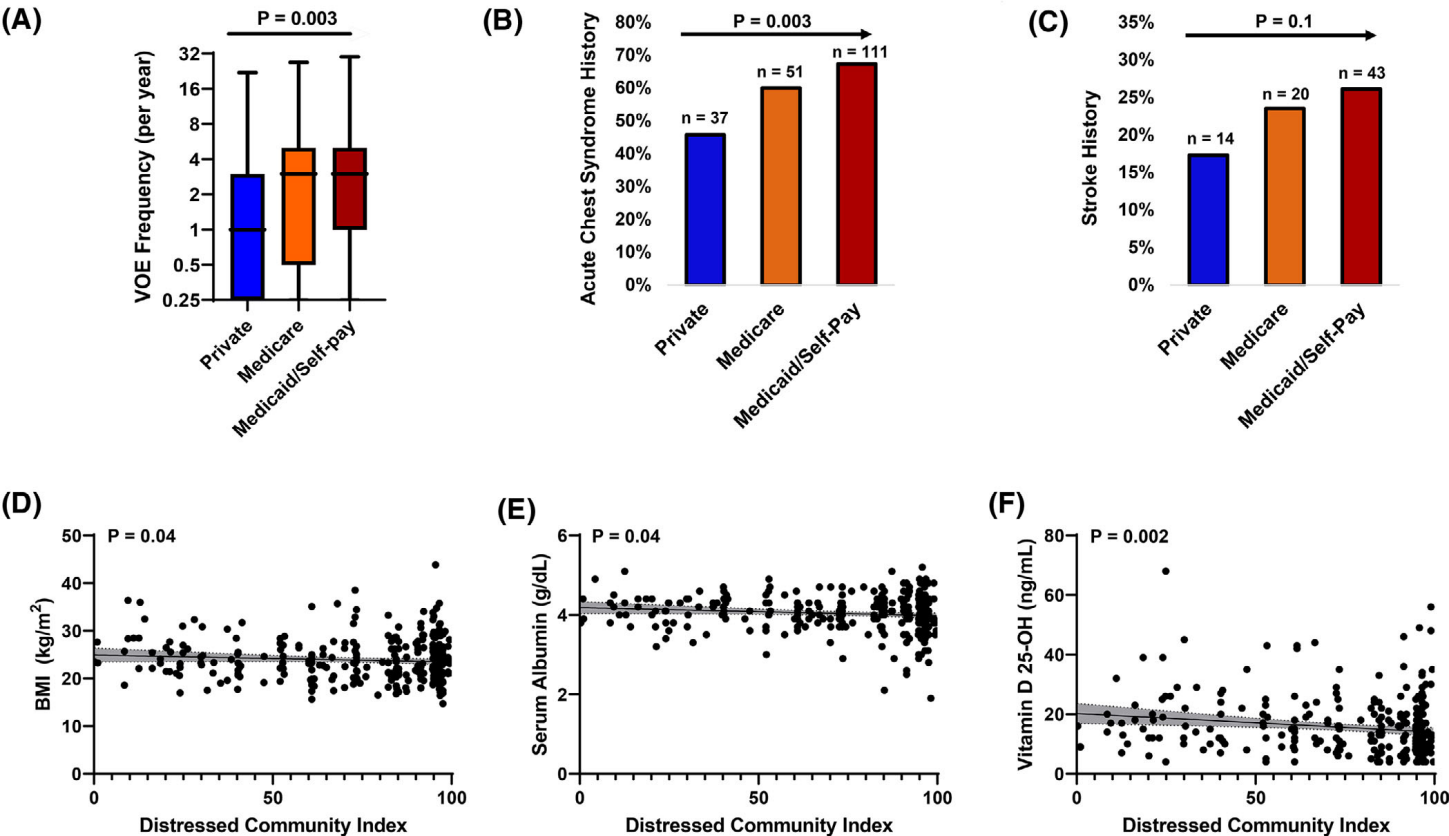
Adults with sickle cell anemia and Medicare or Medicaid/self‐pay insurance had progressively higher rates of **(A)** vaso‐occlusive episodes (VOE) and **(B)** acute chest syndrome history as well as a trend for **(C)** more stroke history compared to those with private insurance. A higher Distressed Community Index (DCI) was associated with lower **(D)** body mass index, **(E)** serum albumin concentration, and **(F)** vitamin D 25‐OH levels in adults with sickle cell anemia.

The median age of the cohort was 31 years (IQR, 24–41 years), 55% were female, 98.5% were African American, and 53% were on hydroxyurea therapy. The median DCI in this cohort was 87 (IQR, 61–96) with 61% of SCA patients living in a distressed tier (DCI ≥ 80) and only 5% living in a prosperous tier (DCI < 20). Fifty percent of the cohort had Medicaid insurance (*n* = 165) or were categorized as self‐pay (*n* = 1), 26% had Medicare insurance, and 24% had private insurance listed as their primary insurance. VOE frequency and acute chest syndrome history were progressively higher while a trend for higher stroke history was observed in SCA patients with Medicare and with Medicaid/self‐pay compared to those with private insurance (Figure 1A–C). The proportion of SCA patients with Medicaid insurance progressively increased with higher DCI tertile (Table 1). SCA patients in progressively higher DCI tertiles were more frequently African American, being treated with hydroxyurea, used tobacco, and had higher serum ferritin concentrations and lower serum vitamin D 25‐OH levels (*p* < 0.05).

**TABLE 1:** Patient characteristics by Distressed Community Index (DCI) score grouped by tertile

	**Low score (DCI < 73; *n* = 110)**	**Middle score (DCI 73 – 95; *n* = 113)**	**High score (DCI > 95; *n* = 109)**	***p*‐Value**
**Age (years)**	31 (26–43)	30 (24–39)	31 (24–42)	0.4
**Females**	43% : 57%	45% : 55%	49% : 51%	0.4
**African American (%)**	106 (96%)	112 (99%)	109 (100%)	0.027
**Insurance Type**				0.006
**Private**	39 (35.5%)	22 (19.5%)	20 (18.3%)	
**Medicare**	29 (26.4%)	32 (28.3%)	24 (22%)	
**Medicaid**	42 (38.1%)	59 (52.2%)	65 (59.6%)	
**Hydroxyurea (%)**	52 (47%)	54 (48%)	69 (63%)	0.018
**Tobacco use (%)**	19 (17%)	21 (19%)	35 (32%)	0.009
**Systolic blood pressure (mm Hg)**	118 (112–128)	120 (111–128)	118 (110–128)	0.5
**Body mass index (kg/m^2^)**	23 (21–26)	23 (21–27)	23 (21–26)	0.3
**WBC count (x 10**[**3**]**/μL)**	9.9 (7.8–12.3)	10 (7.9–12.4)	10.8 (8.1–12.2)	0.2
**Hemoglobin (g/dL)**	8.9 (8.1–9.8)	8.8 (7.9–9.6)	8.8 (7.7–9.6)	0.4
**Platelet count (x 10**[**3**]**/μL)**	393 (295–503)	408 (310–508)	417 (313– 497)	0.4
**Absolute reticulocyte count (x 10**[**3**]**/μL)**	304 (224–426)	310 (224–447)	325 (214–400)	0.6
**Hemoglobin F (%)**	5.6 (2.8–9.7)	6.2 (2.8–9.9)	5.7 (2.7–9.9)	0.8
**LDH (u/L)**	355 (255 – 454)	342 (266–432)	366 (282–482)	0.4
**Ferritin**	419 (121–1068)	497 (214–1396)	656 (204–1730)	0.01
**ALT (u/L)**	23 (18–34)	23 (16–33)	24 (17–31)	0.7
**Serum albumin (g/dL)**	4.1 (3.8–4.3)	4.1 (3.8–4.3)	4.0 (3.8– 4.3)	0.1
**Vitamin D 25‐OH (ng/mL)**	15 (10–23)	13 (8–18)	12 (8–17)	0.004
**eGFR (mL/min/1.73m^2^)**	121 (102–129)	124 (106–132)	122 (97–132)	0.8
**Urine albumin (mg/g creatinine)**	41 (11–210)	34 (10–185)	41 (15–142)	0.7
**VOE frequency (per year)**	2 (1–6)	2 (0–5)	2 (1–4)	0.8
**Acute chest syndrome history**	69 (63%)	68 (60%)	62 (57%)	0.5
**Stroke history**	23 (21%)	31 (27%)	23 (21%)	0.9

Abbreviations: ALT, alanine transaminase; eGFR, estimated glomerular filtration rate; LDH, lactate dehydrogenase; VOE, vaso‐occlusive pain episodes; WBC, white blood cell count.

We did not observe an association between DCI scores and SCA‐related complications, such as VOE frequency, history of acute chest syndrome, or stroke history, on univariate analysis or after adjusting for age, sex, hydroxyurea use, and insurance status (P ≥ 0.07) (**Supplementary Table** **1**). Higher DCI was independently associated with more frequent tobacco use (OR 1.1; *p* = 0.038) as well as with lower body mass index (β = −0.01; *p* = 0.043), serum albumin concentration (β = −0.02; *p* = 0.042), and vitamin D 25‐OH level (β = −0.7; *p* = 0.002) after adjusting for age, sex, hydroxyurea use, and insurance status (Figure 1D–F).

## 4 DISCUSSION

We demonstrate that socioeconomic distress at the community level was associated with tobacco use and with several markers of nutritional status in patients with SCA. Distressed communities are neighborhoods with reduced socio‐economic well‐being which can lead to reduced access to health‐related community resources, such as health care facilities, healthy foods, and recreational activities, as well as an increased risk of exposure to environmental toxins and unhealthy behaviors [9, 10]. In our cohort, 61% of patients with SCA lived in a distressed tier (DCI ≥ 80). This is similar to what has been reported in a cohort of patients with sickle cell disease from England, where 58% lived in the most socio‐economically deprived areas [6]. In another pediatric cohort from Alabama, 45% of children with sickle cell disease lived in the most deprived area [7]. In our study, SCA‐related complications were associated with insurance status but not with DCI. The association of SCA‐related complications with insurance status has been observed in other cohorts of patients with sickle cell disease [11, 12] and our findings suggest that individual‐level indicators of socio‐economic well‐being may be a stronger predictor of these complications than community‐level indicators [13]. A higher DCI score was independently associated with a greater proportion of SCA patients smoking tobacco, which may represent a modifiable risk factor for cardiovascular disease in the general population and for acute chest syndrome in adults with sickle cell disease [14].

We found that a higher DCI score was independently associated with several markers of poor nutrition, including lower body mass index, serum albumin, and vitamin D 25‐OH concentration. Potential reasons for this might include food insecurity and low access to healthy foods. Food insecurity is increased in people with reduced income, low employment, or disability [15]. Risk factors for food insecurity in people with SCA, reported in 30%–46% of other sickle cell disease cohorts [16, 17], are less clear. Although we did not measure food insecurity, our results suggest that community‐level barriers, such as access to healthy foods, may play an important role in the nutritional status of adults with SCA.

Limitations of our study include being cross‐sectional in nature, the narrow distribution of DCI, and lacking additional measures of individual socioeconomic status, such as household income and educational level. Future studies prospectively investigating the effects of socioeconomic distress at an individual and community level will help us better understand the impact of social determinants of health in people with SCA and guide public health strategies to improve health equity for this population.

## AUTHOR CONTRIBUTIONS

SAA, JH, RS, REM, VRG, and JPL designed and performed research and wrote the paper. SLS designed and performed research, analyzed the data, and wrote the paper.

## CONFLICT OF INTEREST STATEMENT

The authors disclose no relevant conflict of interest to this study.

## Supporting information

Table S1 Information

## Data Availability

The data that support the findings of this study are available on request from the corresponding author. The data are not publicly available due to privacy or ethical restrictions.
