# Supplementary material for: Community‐level socioeconomic distress is associated with nutritional status in adults with sickle cell anemia
Source: EJHaem. 2023 Feb 17;4(2):432–6. doi: 10.1002/jha2.661 (PMC10188476; doi:10.1002/jha2.661)
Supplement: Supplementary file 1 — Table S1 Information [file JHA2-4-432-s001.docx]

| **Supplementary Table 1:** Association of Insurance and Distressed Community Index (DCI) Scores with hydroxyurea use and sickle cell anemia-related complications. | | | | | |
| --- | --- | --- | --- | --- | --- |
|  | **Insurance Type** | | **DCI** | | |
|  | **Odds Ratio or β (95% CI)** | **P value** | **Odds Ratio or β (95% CI)** | **P value** |  |
| **VOE Frequency** | +0.86 (0.27 to 1.45) | 0.004 | -0.17 (-0.35 to 0.06 | 0.07 |  |
| **Acute Chest Syndrome** | 1.6 (1.19 – 2.16) | 0.002 | 1.08 (0.99 – 1.2) | 0.09 |  |
| **Stroke** | 1.31 (0.93 – 1.83) | 0.1 | 1.02 (0.92 – 1.13) | 0.7 |  |

VOE, vaso-occlusive pain episodes

Model adjusted for age, sex, hydroxyurea use, insurance type and DCI
